# Effects of Degrees of Aldehyde Modification on Molecular Structures and Properties of Cellulose Nanofiber Cross-Linked Self-Healing Hydrogels

**DOI:** 10.1021/acsomega.5c06391

**Published:** 2025-10-09

**Authors:** Zhi-Yong Wang, Shan-hui Hsu, Shu-Wei Chang, Chia-Ching Chou

**Affiliations:** † Institute of Polymer Science and Engineering, 33561 National Taiwan University, No. 1, Section 4, Roosevelt Rd, Da’an District, 10617 Taipei, Taiwan; ‡ Department of Civil Engineering, 33561 National Taiwan University, No. 1, Section 4, Roosevelt Rd, Da’an District, 10617 Taipei, Taiwan; § Department of Biomedical Engineering, 33561 National Taiwan University, No. 1, Section 4, Roosevelt Rd, Da’an District, 10617 Taipei, Taiwan; ∥ Institute of Applied Mechanics, 33561 National Taiwan University, No. 1, Section 4, Roosevelt Rd, Da’an District, 10617 Taipei, Taiwan

## Abstract

Glycol chitosan (GC) and multifunctional cellulose nanofiber (MCNF) composite hydrogel prepared via Schiff base chemistry have demonstrated excellent self-healing, shear-thinning, and injectable properties, holding promise for various applications. MCNF, a derivative of cellulose nanofibers (CNFs), is introduced with dialdehyde groups through sequential TEMPO-mediated oxidation and periodate oxidation. These aldehyde groups form a dynamic Schiff base linkage with the amine groups of GC chains. While both cellulose and chitosan are biocompatible natural polymers, excessive aldehyde modification of MCNF can disrupt the uniformity of the hydrogel network. To elucidate the underlying molecular mechanism of aldehyde modification of MCNF on affecting the molecular structure of the hydrogel network, here, we performed molecular dynamics simulations on both a single MCNF molecule and a GC/MCNF composite hydrogel with varying degrees of aldehyde modification. For a single MCNF, increased aldehyde modification initially resulted in a more extended molecular conformation, but at higher modification levels (above 15%), the chains became more curled. Correspondingly, the Young’s modulus of MCNF also decreased slightly with increased modification, consistent with the trends in experimental results suggesting reduced mechanical stiffness of the hydrogel due to a loss in intramolecular hydrogen bonding and increased chain flexibility. Among the GC/MCNF hydrogels, the aldehyde content significantly influenced the interaction and network structure. At modification levels below 15%, hydrogen bonding between GC and MCNF was enhanced and evenly distributed, supporting a homogeneous and mechanically robust hydrogel. At higher modification levels, excessive GC–MCNF interactions led to localized aggregation, reduced interconnectivity, and a decline in the tensile modulus. This study provides molecular-level insights into how the aldehyde modification of CNFs affects both the conformation of MCNF and the overall integrity of GC/MCNF hydrogels. These findings offer valuable guidance for optimizing aldehyde-modified CNF-based self-healing hydrogels for biomedical and engineering applications.

## Graphical abstract

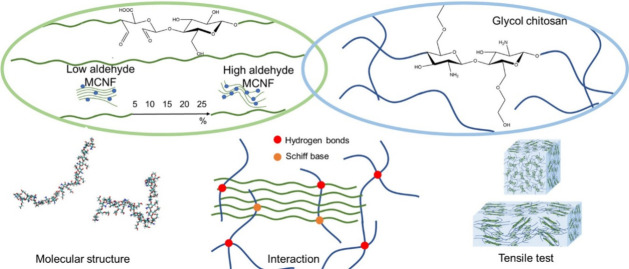

## 1 Introduction

Nanocellulose materials can be broadly categorized into cellulose nanocrystals (CNCs), cellulose nanofibers (CNFs), hairy cellulose nanocrystals (HCNC), and bacterial nanocellulose (BNC), along with their derivatives, based on their size, morphology, and processing methods.^1–5^ These materials originate from cellulose, the most abundant natural polymer on Earth, which is also a renewable biopolymer primarily derived from plants, algae, and bacteria. Nanocellulose exhibits outstanding properties, including a high aspect ratio, large surface area, high mechanical strength, low thermal expansion, low density, and excellent biocompatibility, making it highly promising for various applications.^6–8^ In particular, nanocellulose is often physically incorporated within hydrogels to enhance their mechanical properties.^9^ Recently, dialdehyde nanocellulose, obtained through periodate oxidation, has been used to form self-healing hydrogels via dynamic Schiff base interactions with chitosan-based hydrogels.^10–13^

Self-healing hydrogels have demonstrated great potential in applications such as drug delivery, tissue regeneration, wound healing, tissue adhesives, and bioprinting.^14^ Unmodified chitosan is only water-soluble under acidic conditions, while glycol chitosan with hydrophilic functional groups has better water solubility under neutral conditions. Self-healing hydrogels formed by cross-linking multifunctional cellulose nanofibers (MCNF) with glycol chitosan (GC) exhibit excellent self-healing behavior, along with shear-thinning, injectability, and biodegradability. However, when the aldehyde content in cellulose is excessively high, the hydrogel fails to form a uniform network due to rapid gelation before thorough mixing, leading to structural inhomogeneity.^10^ Similarly, self-healing hydrogels fabricated from hydroxypropyl trimethylammonium chloride chitosan and dialdehyde-modified bacterial cellulose exhibit good self-healing and injectable properties, which are beneficial for wound healing. However, excessive aldehyde content slows down the gelation rate and reduces the compressive modulus.^11^

Molecular dynamics (MD) simulations have been extensively employed to investigate atomic-scale physical phenomena and molecular interaction mechanisms. MD simulations have been applied in studies related to chitosan-based hydrogels^15–18^ and CNFs.^19–26^ Moreover, MD simulations have been used to explore the molecular interaction mechanisms, including the studies related to the cross-linking mechanism of chitosan-phenol hydrogels using dibenzaldehyde poly­(ethylene oxide) (DB-PEO), which correlates with the number of phenol groups in the hydrogel, where intermolecular interactions include van der Waals forces, hydrogen bonding, dynamic Schiff base formation, and π–π stacking.^17^ Additionally, studies on the interaction between dialdehyde cellulose and aliphatic diamines have demonstrated that dynamic Schiff base formation significantly affects the formation of intermolecular hydrogen bonds between cellulose chains.^27^ These studies have demonstrated that MD can assist us to understand the molecular interactions of polymer mechanisms; however, the atomic-level molecular mechanism governing the structure and interactions of hydrogels cross-linked by dialdehyde cellulose remains unclear. In particular, the effect of an excessively high degree of aldehyde modification in nanocellulose on hydrogel properties has yet to be fully elucidated.

In this study, MD simulations were employed to investigate how the aldehyde modification of CNFs influences the molecular structure, hydrogen bonding, and interaction mechanisms at both the single-MCNF level and within GC/MCNF composite hydrogels. In addition, tensile tests were conducted to assess the mechanical behavior of the hydrogels. The molecular interactions between MCNF and GC, along with the key factors governing the network structure and mechanical properties, were explored. These findings aim to provide insights into the design and optimization of cellulose-based self-healing hydrogel systems.

## 2 Materials and Methods

This study consisted of three systems: (1) a single CNF in vacuum, (2) a single MCNF in water, and (3) a GC/MCNF hydrogel system, composed of GC and five CNF chains with various modifications. A five-chain CNF model was constructed based on the X-ray diffraction (XRD) data of cellulose Iβ,^28^ with each chain consisting of 20 glucose monomers. Figure 1A illustrates the synthesis process of MCNF, which involves two sequential functional modifications: TEMPO oxidation^29^ followed by the periodate oxidation.^30,31^ First, the 30% unmodified CNF monomers (Figure 1A, left) were selected for TEMPO oxidation, resulting in TEMPO-oxidized CNF (TCNF; Figure 1A, middle). Subsequent periodate oxidation of the glucose units in TCNF yielded two types of oxidation monomers: (1) dialdehyde cellulose monomer (Figure 1B) and (2) cellulose monomer bearing TEMPO modifications and periodate oxidation (Figure 1A, right). As a result, the MCNF model comprised four types of monomers: unmodified CNF monomer, TEMPO-modified monomer, dialdehyde-modified monomers, and dual-modified monomers. In this study, following experimental conditions,^10^ the five degrees of dialdehyde cellulose modifications were designated as MCNF5, MCNF10, MCNF15, MCNF20, and MCNF25, corresponding to 5, 10, 15, 20, and 25% periodate oxidation levels, respectively. The oxidation sites were evenly distributed throughout the CNF structure, as shown in Figure S1. GC is a linear polysaccharide composed of glucosamine units with glycol group side chains, and the chemical structure monomer is shown in Figure 1C. The degree of polymerization in a GC molecule was set to 20.

**Figure 1:**
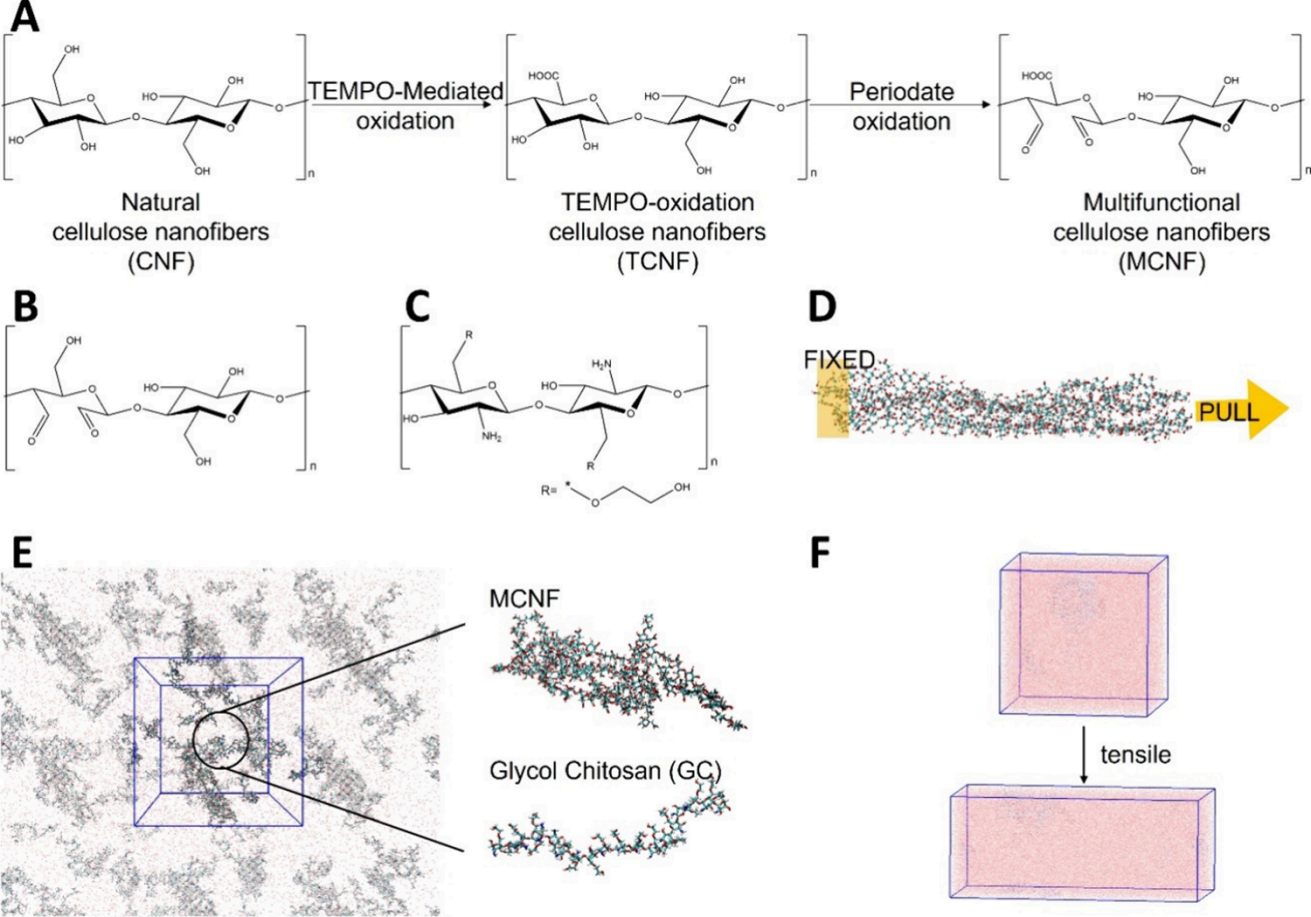
(A) Synthetic process of the MCNFs, involving two sequential functional modifications: TEMPO oxidation followed by periodate oxidation. Chemical structure of (B) dialdehyde-modified monomer by periodate oxidation and (C) GC monomer. (D) Schematic diagram of the model set up during the MCNF tensile test. (E) GC/MCNF composite hydrogel, with single MCNF and GC molecules highlighted on the right. (F) Schematic diagram of the tensile test of GC/MCNF composite hydrogel.

The topological structures of the models were generated using the CHARMM-GUI ligand reader and modeler.^32^ Visualization and analysis of the simulation results were conducted using Visual Molecular Dynamics (VMD)^33^ to compute various physical properties of the polymer systems. The CHARMM36 force field,^34,35^ as shown in eq 1 was applied to describe the interaction between atoms. The potential energy, *E*_total_, is the sum of bonded and nonbonded terms, expressed as a function of atomic coordinates. Bonded terms, including bond (*b*), valence angle (θ), dihedral angle (φ), improper dihedral angle (Ø), and Urey–Bradley equilibrium (*S*) terms, with the Urey–Bradley terms, are typically employed under specific conditions. In the equation, *K* represents the respective force constants: *K*_b_ for bonded force, *K*_θ_ for angle force, *K*_φ_ for dihedral force, *K*_Ø_ for improper angle force, and *K*_UB_ for Urey–Bradley equilibrium. Nonbonded terms include van der Waals (vdW) and electrostatic interactions, which focus on the interaction of the Lennard-Jones 6–12 (LJ 6–12) and between point charges (atom *i* and *j*) terms. In the nonbonded part, ε is the well depth, *R* is the radius of LJ terms used to explain vdW interactions, *r* is the distance between two atoms, and is the partial charge carried by each atom.

$$E_{\mathrm{total}}=\sum_{\mathrm{bonds}}K_{b}{\left(b-b_{0}\right)}^{2}+\sum_{\mathrm{angles}}K_{\theta }{\left(\theta -\theta_{0}\right)}^{2}+\sum_{\mathrm{dihedrals}}K_{\varphi }\left[1+\cos\left(n\varphi -\delta \right)\right]+\sum_{\mathrm{improper}}K_{Ø}{\left(Ø-{Ø}_{0}\right)}^{2}+\sum_{\mathrm{Urey}-\mathrm{Bradley}}K_{UB}{\left(S-S_{0}\right)}^{2}+\sum_{\mathrm{vdW}}\varepsilon_{ij}\left[{\left(\frac{R_{\min.ij}}{R_{ij}}\right)}^{12}-2{\left(\frac{R_{\min,ij}}{R_{ij}}\right)}^{6}\right]+\sum_{\mathrm{electrostatic}}\frac{q_{i}q_{j}}{4\pi \varepsilon_{0}r_{ij}}$$ 1

The single MCNF system was used to better understand the effect of aldehyde modification on the intrinsic properties of MCNF and to know how the GC influences MCNF behavior in the hydrogel system. In the single MCNF simulations, first, an NVT equilibrium simulation is performed in a vacuum at 300 K for 15 ns to obtain an MCNF model with a preliminary stable state. Then, the CNF was placed in a rectangular water box of approximately 55 × 55 × 130 Å. After energy minimization, the system was equilibrated at 1 atm and 300 K under NPT conditions for 10 ns, followed by NVT equilibration for 30 ns at the same temperature. The simulation was performed using NAMD 3.0.^36^ and the time step was set to 2 fs. The final 5 ns of the simulation were used to compute the average values and calculate the standard deviations using the block average. The model was further subjected to MCNF tensile simulations in water.

A steered MD simulation (SMD)^37^ was employed to study the tensile behavior of a single MCNF in water using NAMD 3.0.^36^ In Figure 1D, the MCNF had one end (the first residue of each chain) fixed in position, while the other end was pulled at a constant velocity of 0.002 Å/ps. The time-dependent stress and strain data obtained from these simulations provided insights into the mechanical properties of MCNFs.

In the hydrogel system, the composition of MCNF and GC was determined based on the weight ratios used in experimental hydrogel preparation.^10^ A MCNF­(Aldehyde modification degree 5 to 25%) and 12 GC molecules were randomly placed in a water box. Figure 1E (left) shows the hydrogel model formed by the MCNF and GC mixture. The single MCNF and GC molecules are highlighted in Figure 1E (right). The concentration of GC in the hydrogel system was maintained at 4.5 wt %, while the MCNF concentration was set at 1.5 wt %. After energy minimization, the hydrogel system was equilibrated at 1 atm and 300 K under NPT conditions for 20 ns, followed by NVT equilibration at the same temperature for 60 ns. The simulation was performed using NAMD 3.0.^36^ and the time step was set to 2 fs. The final 10 ns of the simulation were used to compute the average values and calculate the standard deviations using the block average. The equilibrated hydrogel models were then subjected to tensile simulations, as shown in Figure 1F. The system was subjected to tensile deformation in the *z* direction at a strain rate of 5 × 10^9^/s with a time step of 1 fs. The periodic boundary conditions were imposed in three directions to avoid any surface effects. The stress and strain data obtained from these simulations provided insight into the mechanical properties of the hydrogel. The mechanical tests of GC/MCNF composite hydrogel were simulated using LAMMPS (version 2 Aug 2023).^38^

For cellulose nanofibers and polymer hydrogels, hydrogen bonds are very important analytical bases that can help us understand the intramolecular and intermolecular interactions in the system. The conditions of eq 2 are used to determine hydrogen bonds.^39^

$$\mathrm{Hydrogen}\;\mathrm{bond}\;=\{\begin{array}{ll} 1, & \left(\begin{array}{l} \mathrm{dis}\tan\mathrm{ce}\left(\mathrm{donor}\;...\;\mathrm{acceptor}\right)\leq \;3.5\;Å\\ \mathrm{and}\;\mathrm{angle}\left(H-\mathrm{donor}\;...\;\mathrm{acceptor}\right)\leq \;30^{{}^{\circ}} \end{array}\right)\\ 0, & \mathrm{otherwise} \end{array}$$ 2

The radius of gyration and the end-to-end distance are both common parameters that describe the structure of a polymer in space. The radius of gyration can be used to determine the stretch of a polymer chain. It is calculated by taking the square root of the average of the squares of the distances from each atom on the molecular chain to the center of mass of the molecular chain, as shown in eq 3. The end-to-end distance is the distance between the two atoms at the end of the molecular chain and can be used to determine the length of the polymer chain. By combining the two parameters, we can know the molecular conformation of the polymer chain, such as the degree of curling, softness, and flexibility. End-to-end distances and radii of gyration were used to evaluate the molecular structure of MCNF and GC, providing insight into how chemical modifications influence their conformation.

$$R_{G}=\frac{1}{N}\sum_{N}^{i=1}(|{\vec{r}}_{i}-\vec{r_{\mathrm{cm}}}|{)}^{2}$$ 3

## 3 Results and Discussion

### 3.1 Effects of Aldehyde Modification on the Properties of Single MCNF Molecules

#### 3.1.1 Molecular Structures of Single MCNF Molecules

First, we analyzed the molecular structure of a single MCNF in water. The equilibrium state of the system was evaluated by using root-mean-square deviation (RMSD). To further confirm equilibrium, the time-dependent variations in end-to-end distance and radius of gyration were examined, as shown in Figure S2. These two structural parameters exhibited consistent trends as the degree of aldehyde modification increased, as shown in Figure 2A. When the modification was below 15%, an increase in modification led to a longer end-to-end distance and a greater radius of gyration, indicating a transition toward a more extended structure. However, when the modification exceeded 15%, both the end-to-end distance and radius of gyration decreased. A more detailed analysis of the end-to-end distance is shown in Figure S3.

**Figure 2:**
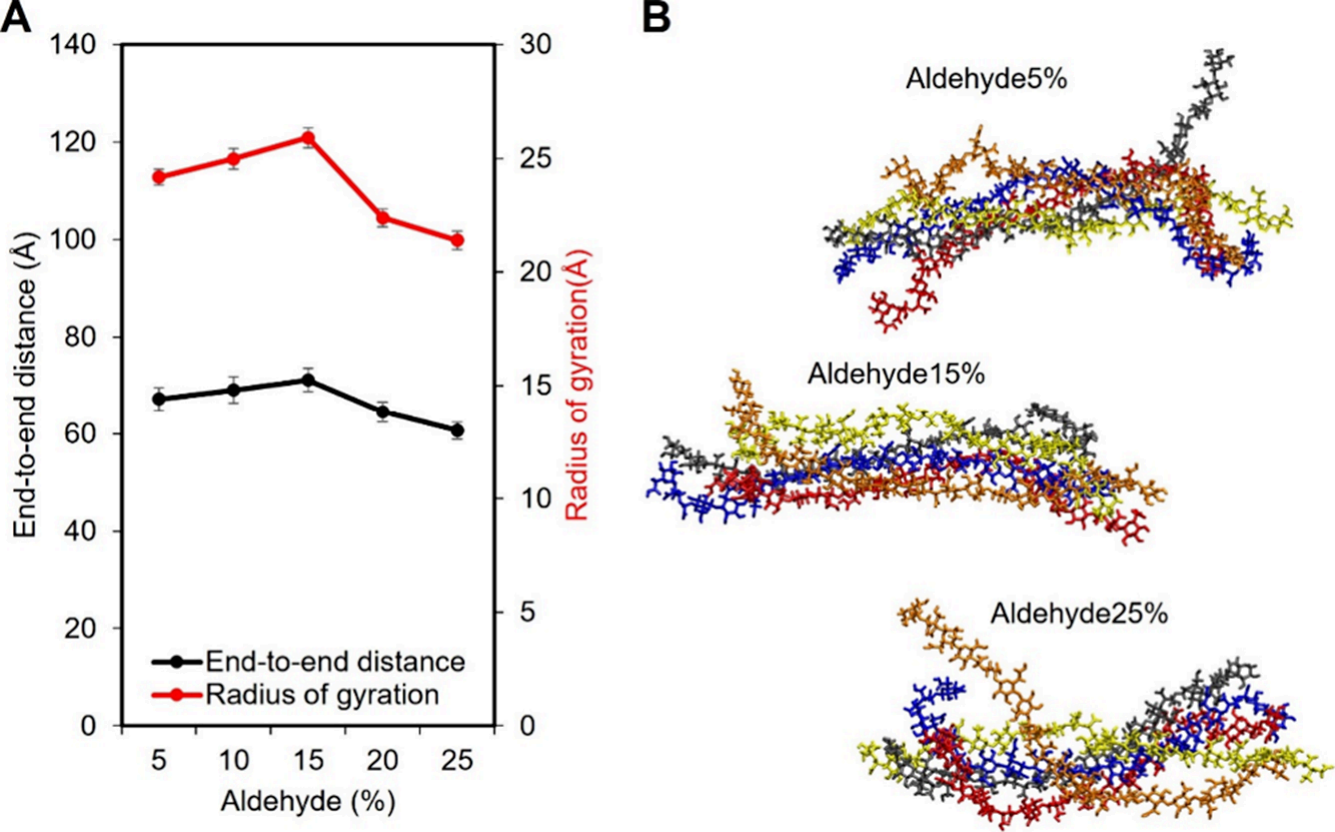
(A) Radius of gyration and end-to-end distance of a single MCNF in water. (B) Snapshots of MCNF5, MCNF15, and MCNF25 molecular structures.

Figure 2B further illustrates the effect of aldehyde modification on the molecular conformation of MCNF. The snapshots show that the spatial arrangement of the MCNF molecular chains changes with increasing modification. At 5% modification, the terminal regions of some MCNF5 chains partially detached from the main structure and exhibit curling, while the central region remained relatively straight. At 15%, the entire MCNF15 adopted a more extended and straightened conformation. However, at 25% modification, molecular chain reverted to a more curled state, and the overall structure becomes more compact once again.

#### 3.1.2 Hydrogen Bonding and Mechanical Properties of Single MCNF Molecules

To understand how aldehyde modifications influence the intermolecular and intramolecular interactions of a single MCNF, hydrogen bond analysis was performed. As shown in Figure 3A, a decrease in the total number of hydrogen bonds primarily results from a reduction in intramolecular hydrogen bonds, while the number of intermolecular hydrogen bonds remains nearly unchanged. After aldehyde modification, the number of hydrogen bonds involving aldehyde groups increases gradually at lower modification levels and rises more substantially once the modification reaches 15%.

**Figure 3:**
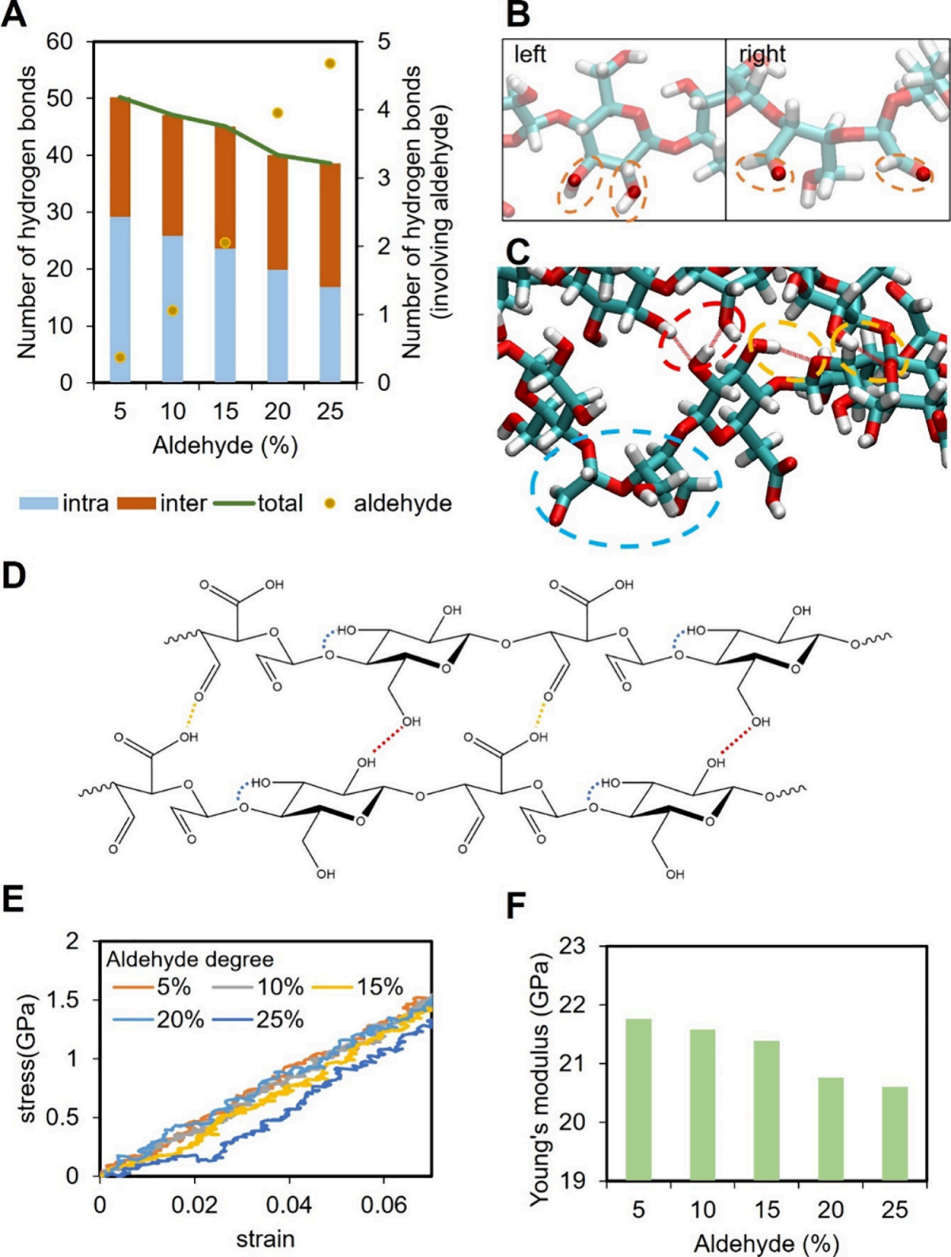
Hydrogen bonding and mechanical properties of single MCNF systems with varying degrees of aldehyde modification. (A) Number of total, intramolecular, and intermolecular hydrogen bonds in a single MCNF system as a function of aldehyde modification. (B) Snapshots of the molecular structures of the MCNF monomer: the orange dashed line highlights the positions of the hydroxyl groups before modification (left) and the aldehyde groups after modification (right), showing increased chain flexibility due to glucose ring-opening. (C) Snapshots of the molecular structure of MCNF20. The blue dashed line encircles the intramolecular hydrogen bonds, the red dashed line encircles the intermolecular hydrogen bonds, and the yellow dashed line encircles the aldehyde-modified monomer. (D) Visualization of hydrogen bonding types in MCNF structures: the intermolecular (red), intramolecular (blue), and aldehyde-modified (yellow) hydrogen bonds. (E) Stress–strain curves of single MCNF chains under tension at various aldehyde modification levels. (F) Young’s modulus of MCNF as a function of aldehyde modification, showing a slight decrease in stiffness with increasing modification.

We next examined how aldehyde modifications affect the molecular structures underlying these changes. Figure 3B illustrates the enhanced mobility of the molecular chains following aldehyde modification. The orange dashed line highlights the positions of the hydroxyl groups before modification (left) and the aldehyde groups after modification (right). Aldehyde modification, involving glucose ring-opening, increases the chain flexibility, facilitating the formation of hydrogen bonds involving aldehyde groups and intermolecular hydrogen bonds. Figure 3C shows the simulation snapshot of the molecular structure of MCNF20. In the figure, blue dashed lines indicate intramolecular hydrogen bonds, red dashed lines denote intermolecular hydrogen bonds, and yellow dashed lines highlight aldehyde-modified monomers. Although aldehyde modification decreased the number of hydrogen bond donors, the increased mobility of the molecular chains enhances the possibility of intermolecular hydrogen bond formation, resulting in a constant number of intermolecular hydrogen bonds. Again, the distribution of hydrogen bonds of the MCNF, including intermolecular (red), intramolecular (blue), and aldehyde groups (yellow), is illustrated in Figure 3D. A significant portion of intramolecular hydrogen bonds were formed between hydroxyl and ether groups within the same or adjacent monomers. Upon oxidation to aldehyde groups, the number of hydrogen bond donors was reduced, leading to a decline in intramolecular hydrogen bonding. However, these aldehyde groups can form hydrogen bonds with hydroxyl groups on neighboring molecules, providing an alternative mechanism for intermolecular hydrogen bond formation.

As a result, at lower degrees of aldehyde modification (for example, 5%), MCNF5 adopted a more curled structure due to a high number of intramolecular hydrogen bonds. In contrast, at higher degrees of modification (MCNF20 and MCNF25), the excessive formation of intermolecular hydrogen bonds involving aldehyde groups led to more compact and curled conformations. Intermolecular hydrogen bonding serves as the primary driver of conformational contraction; however, without sufficient chain flexibility, these bonds cannot be established. Therefore, both hydrogen bonding and flexibility are essential, with flexibility playing a dominant role in this mechanism.

To evaluate the effect of aldehyde modifications on the mechanical properties of MCNF, tensile tests were performed. Figure 3E presents the stress–strain curves of single MCNFs with varied degrees of aldehyde modifications, from 0 to 7% strain. As shown in Figure 3F, Young’s modulus decreased slightly from 21.767 GPa for MCNF5 to 20.608 GPa for MCNF25, indicating a reduction in mechanical stiffness with increasing aldehyde modifications. The modulus values fall within the experimentally reported ranges for cellulose nanofibers (12.6 to 37.5 GPa).^40,41^ The decrease in modulus suggests that aldehyde modification weakens the mechanical strength of MCNF, which can be attributed to two factors: (1) a reduction in the number of hydrogen bonds and (2) an increase in chain flexibility due to glucose ring-opening induced by aldehyde formation, consistent with the observation form previous studies.^42^

In addition to the simulations in aqueous environments, the tensile tests of CNFs in a vacuum were also studied, and the results are provided in Figure S4. The results are consistent with reported ranges for the tensile modulus of cellulose nanofibers, including experimental values (105–220 GPa),^43,44^ MD simulations (107.8–161 GPa),^19,20,45,46^ and first-principles calculations (206 GPa).^47^

### 3.2 Effect of Aldehyde Modification of MCNF on the Properties of GC/MCNF Composite Hydrogels

#### 3.2.1 Molecular Structures of GC/MCNF Composite Hydrogels

The equilibrium state of the GC/MCNF composite hydrogel model was first assessed using RMSD, followed by the analysis of end-to-end distance and radius of gyration over time to confirm further equilibration, as shown in Figure S5. After reaching equilibrium, the simulated density of the system stabilized around 1 g/cm^3^, which is consistent with the reported values in experimental studies,^48,49^ as shown in Figure S6, indicating the validity of our simulation approach for investigating the spatial structure and molecular interactions within the composite hydrogel system.

The averaged end-to-end distance and radius of gyration of GC after equilibration are shown in Figure 4A. Across all models, the GC exhibited minimal variation, indicating structural stability irrespective of the MCNF modification. In contrast, Figure 4B shows that MCNFs exhibited a trend similar to that observed in a single MCNF system. When the degree of aldehyde modification is lower than 15%, increasing modification led to a more extended and straightened MCNF structure. However, as modification increased beyond 15%, the molecular chains became more curled again. A more detailed analysis of the end-to-end distance is shown in Figure S7 and S8.

**Figure 4:**
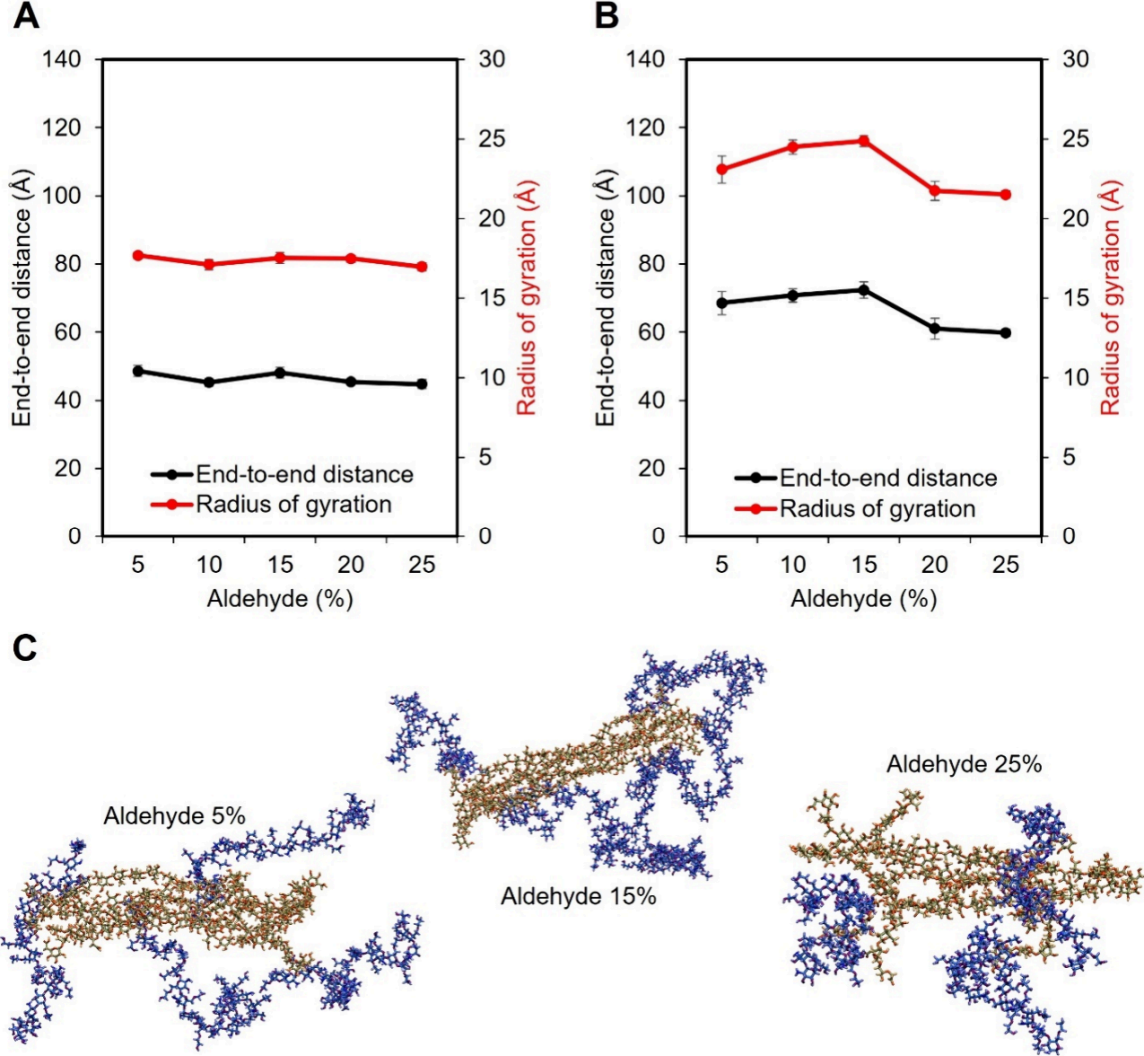
Averaged radius of gyration and end-to-end distance of (A) GC and (B) MCNFs in the hydrogel model. (C) Snapshots of GC/MCNF composite hydrogels at different degrees of aldehyde modification (5, 15, and 25%). Only GC molecules that are in close interaction with the MCNF are displayed, where MCNF is colored orange and GC is colored in blue.

Figure 4C presents snapshots of MCNF molecular structures surrounded by GC molecules in the GC/MCNF composite hydrogel at various degrees of aldehyde modification. The molecular structures of MCNF in the composite hydrogel closely resemble those observed in the single MCNF system in water. At 5% modification, several terminal chains of MCNF were entangled with GC, partially detached from the main structure and exhibited curling at the ends, while the central region remains relatively straight. At 15%, the MCNF adopted a more uniformly extended conformation. However, at 25% modification, the entire MCNF structure became more curled, with some terminal segments separating from the central chains and interacting more strongly with GC. These observations were consistent with the conformational changes observed in single MCNF systems. Furthermore, at 5 and 15% aldehyde modification, each GC chain interacting with MCNF also engaged with other components in the system, such as neighboring GC molecules or water (not shown in the figure). This interaction may promote the formation of a more uniform and interconnected hydrogel network. In contrast, at 25% modification, some GC chains appeared to be bound to the MCNF surface or curled themself, limiting their availability to interact with other components. This reduced interaction capacity may contribute to a less homogeneous and more locally aggregated hydrogel structure.

#### 3.2.2 Hydrogen Bonding and Mechanical Properties of GC/MCNF Composite Hydrogels

To investigate the effect of MCNF aldehyde modification on the interactions within the hydrogel network, we analyzed the hydrogen bonds in the composite hydrogel model. The number of intramolecular hydrogen bonds within the GC remained relatively unchanged across different degrees of modification. Figure 5A shows the number of hydrogen bonds, including intermolecular hydrogen bonds within GC, hydrogen bonds between GC and MCNF, and intramolecular and intermolecular hydrogen bonds within MCNF. A schematic summary of the changes in hydrogen bond patterns with increasing aldehyde modification is plotted in Figure 5A right.

**Figure 5:**
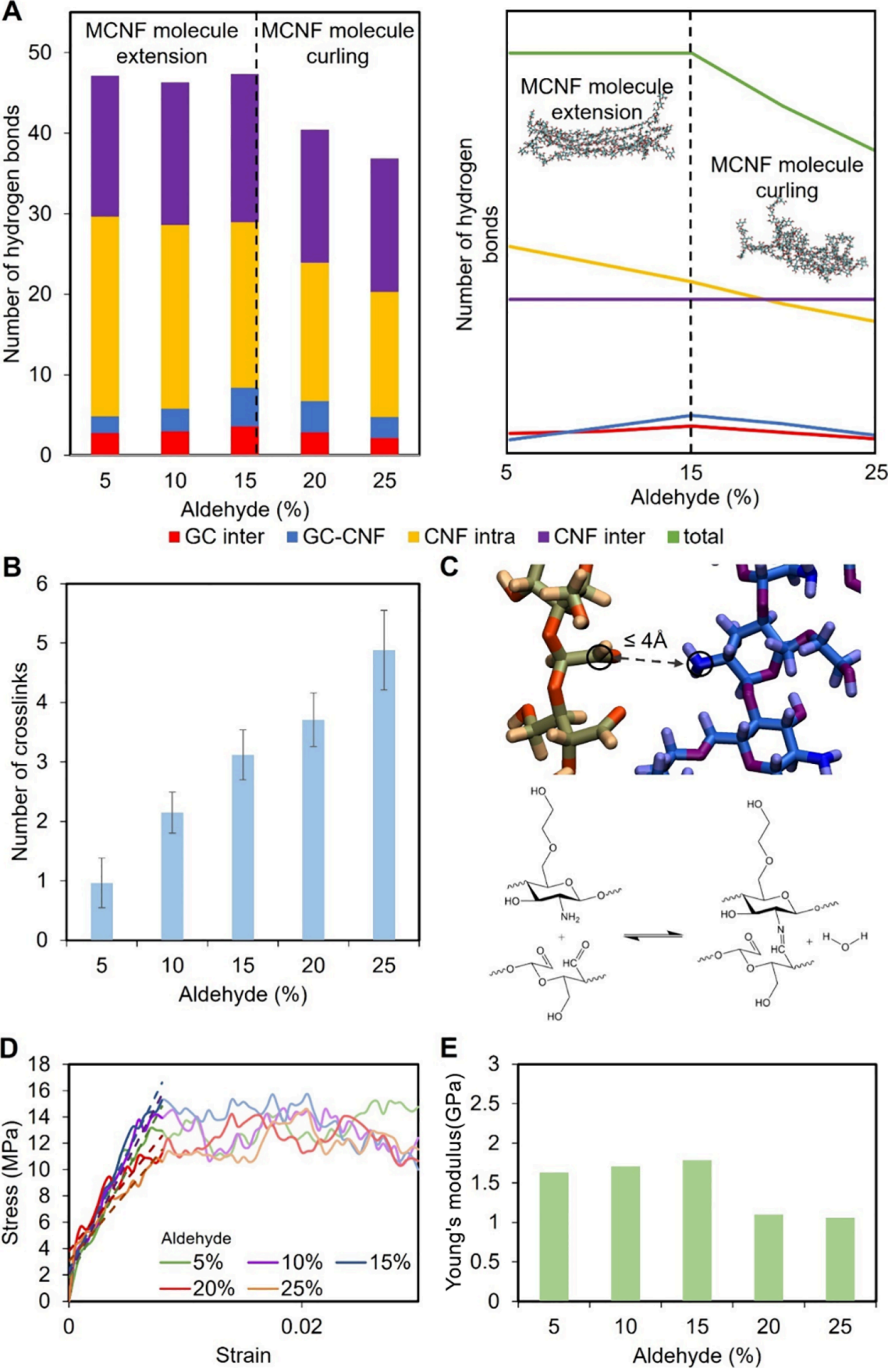
(A) Left: number of hydrogen bonds in the GC/MCNF hydrogel system at different degrees of aldehyde modification. Categories include hydrogen bonds between GC molecules, hydrogen bonds between GC and MCNF, and intra- and intermolecular hydrogen bonds within MCNF. Right, schematic illustration of hydrogen bonding trends in the hydrogel system as a function of increasing aldehyde modification. Hydrogen bond interactions between GC and MCNF and between GC molecules initially increase (up to 15% modification) and then decrease at higher modification levels, while overall MCNF hydrogen bonding declines with increased aldehyde content. (B) Probability of dynamic Schiff base cross-links, it is determined by the number of aldehyde groups within 4 Å of an amine group. (C) MCNF is shown in orange, and glycol chitosan is shown in blue. Chemical structures depict dynamic Schiff base cross-linking. (D) Stress–strain curves of GC/MCNF composite hydrogels with different degrees of aldehyde modification under uniaxial tensile testing. The slope of each dashed line was represented the calculate Young’s modulus. (E) Young’s modulus of GC/MCNF composite hydrogels calculated from the linear region (black dashed lines) of the stress–strain curves in Figure 5D. Modulus increases with modification from 5 to 15%, and then decreases at higher levels (20 and 25%) due to reduced hydrogen bonding and network integrity.

The total number of hydrogen bonds involving MCNF decreases with an increase in aldehyde modification, consistent with the observation from a single MCNF simulation in water. Notably, the total hydrogen bond count remains relatively stable up to 15% modification but decreases beyond 15%. Both GC–MCNF and GC–GC intermolecular hydrogen bonds initially increase with aldehyde modification, peaking around 15%, and then decrease at higher modification levels. These hydrogen bonding patterns aligned with the molecular conformations observed in our simulations, as previously shown in Figure 3C. In particular, MCNF15 exhibited a favorable structural arrangement in which GC chains interacting with MCNF also engaged with surrounding GC molecules or water, facilitating extensive intermolecular bonding and promoting the formation of a more interconnected hydrogel network.

We counted the number of possible Schiff base cross-links to determine their impact on the hydrogel system, as shown in Figure 5B. Figure 5C shows the number of aldehyde groups within 4 Å of an amine group and depicts the dynamic Schiff base cross-links using chemical structures. The results indicate that the occurrence of Schiff bases is low. We then modeled and simulated Schiff bases, with the results shown in Figure S9. The Schiff bases showed a minimal effect on the end-to-end distance of the MCNF and hydrogen bonds in GC/MCNF systems. Both measurements in the bonded systems remained comparable to those observed in the nonbonded systems. It is important to note, however, considering the limited length scale and relatively small molecular weight of the model, the simulations may capture only a restricted number of Schiff base cross-links compared with experimental conditions.

Figure 5D shows the stress–strain curves of GC/MCNF hydrogels with varying degrees of aldehyde modification during tensile testing, and the corresponding Young’s moduli are calculated from the initial linear region, as shown in Figure 5E. Compared with the experiment, the hydrogel modulus is higher because it was performed at a higher strain rate (due to limitations in computing power), and previous experiments or simulations have shown that increasing the strain rate leads to an increase in modulus.^50–53^ The mechanical strength of hydrogels increases from 5 to 15% aldehyde modification but decreases at 20 and 25%. The enhanced strength in the 5–15% range correlated with straighter MCNF conformations and a higher number of GC–MCNF hydrogen bonds, which promoted a more interconnected hydrogel network. In contrast, at 20 and 25% modification, the MCNF chains became more curled, and the reduced number of hydrogen bonds between MCNF and GC, as well as between GC molecules, may result in a less cohesive network, ultimately lowering the mechanical strength of the hydrogel.

## 4 Conclusions

This study investigated the molecular mechanism for the effects of aldehyde modification on single MCNF molecules and GC/MCNF composite hydrogels using MD simulations. For single MCNF molecules, the results demonstrated that increasing the degree of aldehyde modification initially led to a more extended, linear conformation, but beyond 15% modification, the chains adopted a more curled structure. The tensile strength of single MCNF was comparable to the experimental data and showed a slight decrease with increasing aldehyde modification. The effect of aldehyde modification on the molecular interactions in the GC/MCNF composite hydrogels was also explored. Structural analysis revealed that aldehyde modification significantly affected the conformation and the intermolecular interactions within hydrogels.

As summarized in Figure 6, when the degree of aldehyde modification ranges from 0 to 15%, MCNF chains become straighter and the interaction between GC and MCNF is more evenly distributed, resulting in a more homogeneous network and enhanced tensile modulus. In contrast, at the higher degree of modification (15–25%), the MCNF chains became more curled, and the interaction with GC became more localized and uneven, leading to a less uniform hydrogel structure and reduced mechanical strength. Overall, this molecular level study of cellular nanofiber-based hydrogels provides crucial insights into the role of chemical modifications in tuning intermolecular interactions and hydrogel performance. The findings contribute to a deeper understanding of CNF-polymer interactions and support the rational design of high-performance hydrogel materials through targeted molecular engineering strategies.

**Figure 6:**
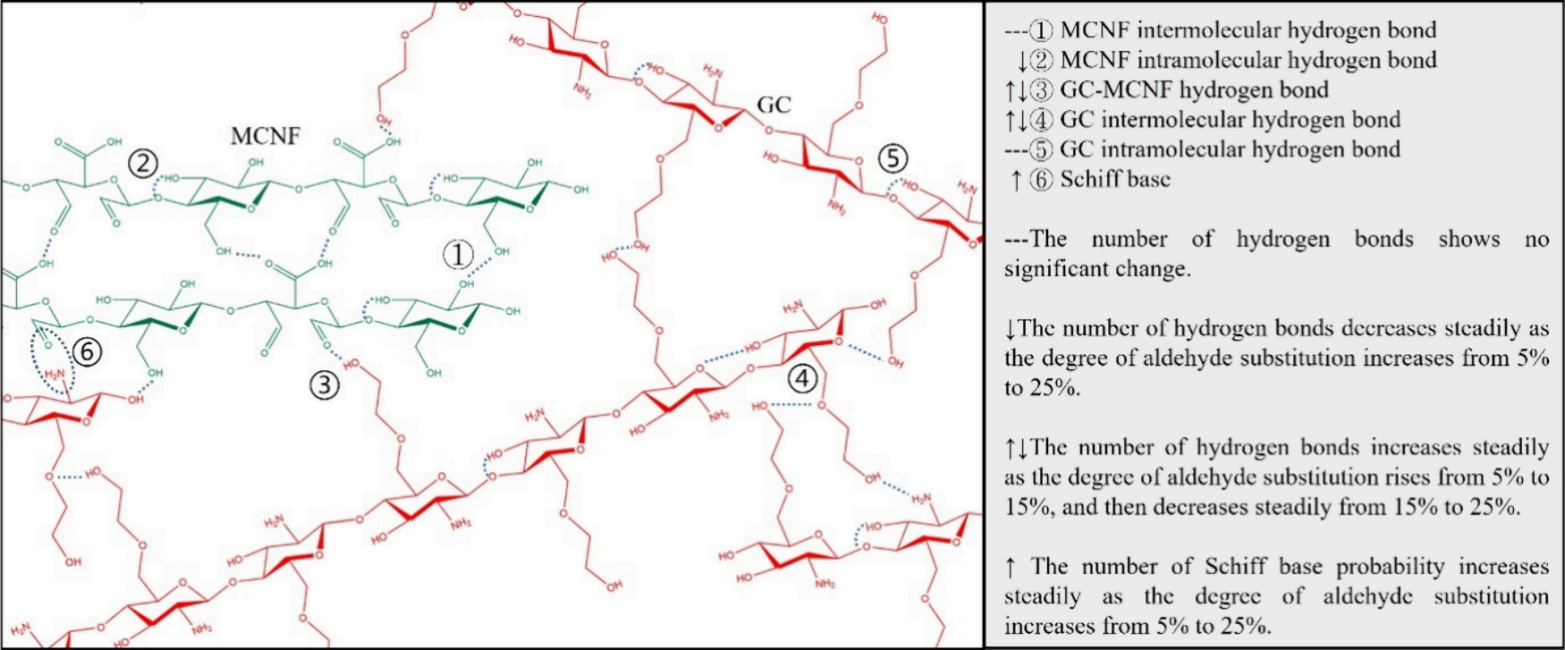
Schematic representation of the interactions in a GC/MCNF composite hydrogel.

## Supplementary Material



## Notes

Details of cellulose nanofiber modification sites, details of equilibrium assessment for the single MCNF system, detailed analysis of the end-to-end distance for the single MCNF system, simulation results of CNFs in vacuum, detail of the assessment of equilibrium and comparison of the densities of hydrogel systems, detailed analysis of the end-to-end distance for the GC/MCNF hydrogel systems, and comparison of results before and after Schiff base modeling (PDF)
