# Supplementary material for: Effects of Degrees of Aldehyde Modification on Molecular Structures and Properties of Cellulose Nanofiber Cross-Linked Self-Healing Hydrogels
Source: ACS Omega. 2025 Oct 9;10(42):49997–50006. doi: 10.1021/acsomega.5c06391 (PMC12573001; doi:10.1021/acsomega.5c06391)
Supplement: Supplementary file 1 [file ao5c06391_si_001.pdf]

Supporting Information

Effects of Degrees of Aldehyde Modification on Molecular Structures and Properties of Cellulose Nanofiber Cross-Linked Self-Healing Hydrogels

Zhi-Yong Wang[1], Shan-hui Hsu[1], Shu-Wei Chang[2 3], Chia-Ching Chou[4]

[1]Institute of Polymer Science and Engineering, National Taiwan University, Roosevelt Road No. 1, Sec. 4, 10617 Taipei, Taiwan

[2]Department of Civil Engineering, College of Engineering, National Taiwan University, Roosevelt Road No. 1, Sec. 4, 10617 Taipei, Taiwan

[3]Department of Biomedical Engineering, College of Engineering, National Taiwan University, Roosevelt Road No. 1, Sec. 4, 10617 Taipei, Taiwan

[4]Institute of Applied Mechanics, College of Engineering, National Taiwan University, Roosevelt Road No. 1, Sec. 4, 10617 Taipei, Taiwan

[Content]

I. Details of cellulose nanofiber modification sites.

II. Details of equilibrium assessment for the single MCNF system.

III. Detailed analysis of the end-to-end distance for the single MCNF system.

IV. Simulation results of CNFs in vacuum.

V. Detail of the assessment of equilibrium and comparison of the densities for GC/MCNF hydrogel systems.

VI. Detailed analysis of the end-to-end distance for the GC/MCNF hydrogel systems.

VII. Comparison of results before and after Schiff base modeling.

I. Detail of cellulose nanofiber modification sites.

Our cellulose nanofiber (CNF) model underwent two types of chemical modifications. In experimental studies, TEMPO oxidation enhances the water solubility of CNFs, while periodate oxidation generates aldehyde groups that can form dynamic Schiff bases with the amine groups of chitosan. In the model, TEMPO oxidation was applied first, followed by further modification to obtain MCNF. The modification sites of the two oxidation processes were independent of each other. Both types of modifications were evenly distributed among the five molecular chains and, after combination, were uniformly dispersed throughout the entire MCNF structure.

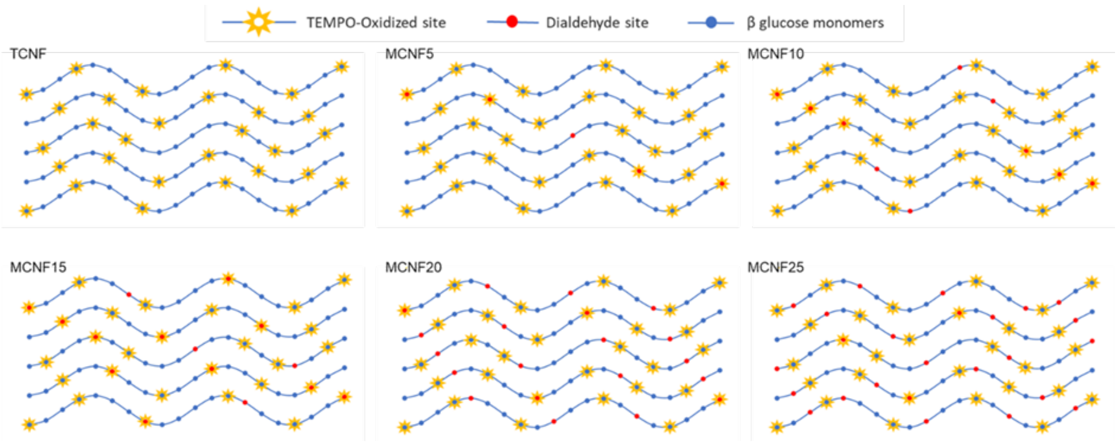

Fig. S1. Schematic diagram of the position distribution of cellulose nanofibers with different modification degrees after two modifications.

II. Details of equilibrium assessment for the single MCNF system.

The equilibrium state of the system was determined using root-mean-square deviation (RMSD). As shown in Figures S2(A), MCNF in water equilibrated after 35 ns. Additionally, the variations in end-to-end distance and radius of gyration over time for CNFs with different degrees of modification were analyzed, as illustrated in Figures S2(B) and S2(C). Since significant fluctuations in CNFs ceased after 35 ns in water, we conclude that the models reached equilibrium.

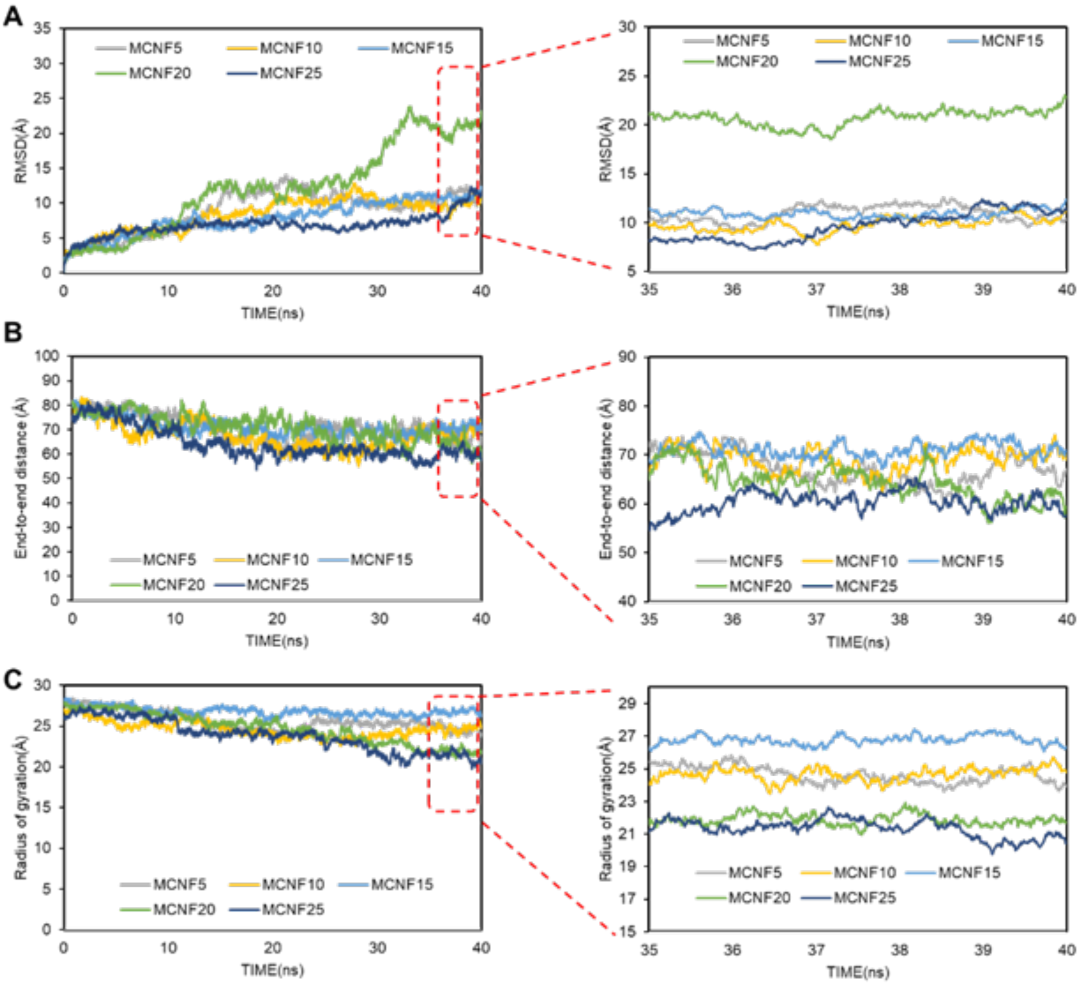

Fig. S2. (A) RMSD, (B) end-to-end distance, and (C) radius of gyration of a single cellulose nanofiber system in water as a function of the degree of modification. The left figure shows the changes in these values during the simulation, and the right figure shows the changes during the equilibrium stage.

III. Detailed analysis of the end-to-end distance for the single MCNF system.

A more detailed analysis of the end-to-end distance of the single MCNF System is conducted to understand the significance of the results better. Under the same degree of aldehyde substitution, the results of each chain of MCNF will not be much different. Error bars are standard deviations, calculated from 500 data points over 5 ns in the analysis.

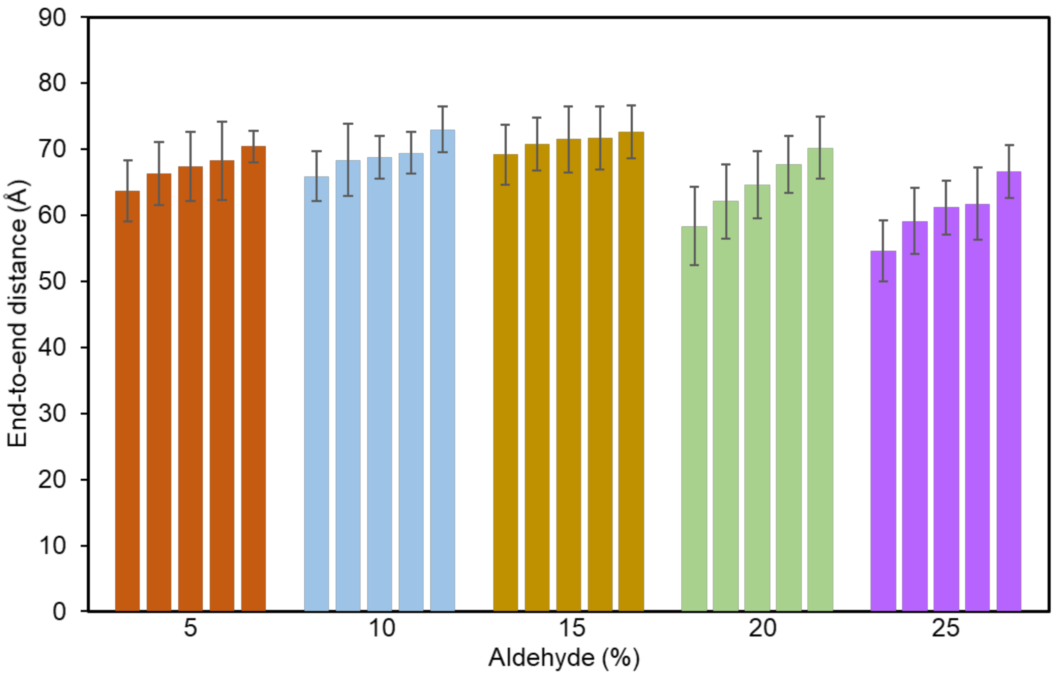

Fig. S3. The end-to-end distance of each MCNF molecular chain for the Single MCNF System.

IV. Simulation results of CNFs in vacuum.

Figure S4(A) is a stress-strain curve of cellulose nanofibers from 0 to 10% strain. The results indicate that both TEMPO oxidation and aldehyde substitution lead to a reduction in mechanical strength. The Young's modulus continuously decreased from 131.38 GPa in unmodified CNFs to 109.83 GPa at 25% aldehyde group substitution, as shown in Figure S4(B). Figure S4(C) shows the hydrogen bond composition of CNF in vacuum equilibrium. The trends of tensile strength and hydrogen bonding changes are similar to those in water. The results are consistent with reported ranges for the tensile modulus of cellulose nanofibers, including experimental values (105–220 GPa)[43, 44], MD simulations (107.8–161 GPa)[19, 20, 45, 46], and first-principles calculations (206 GPa)[47].

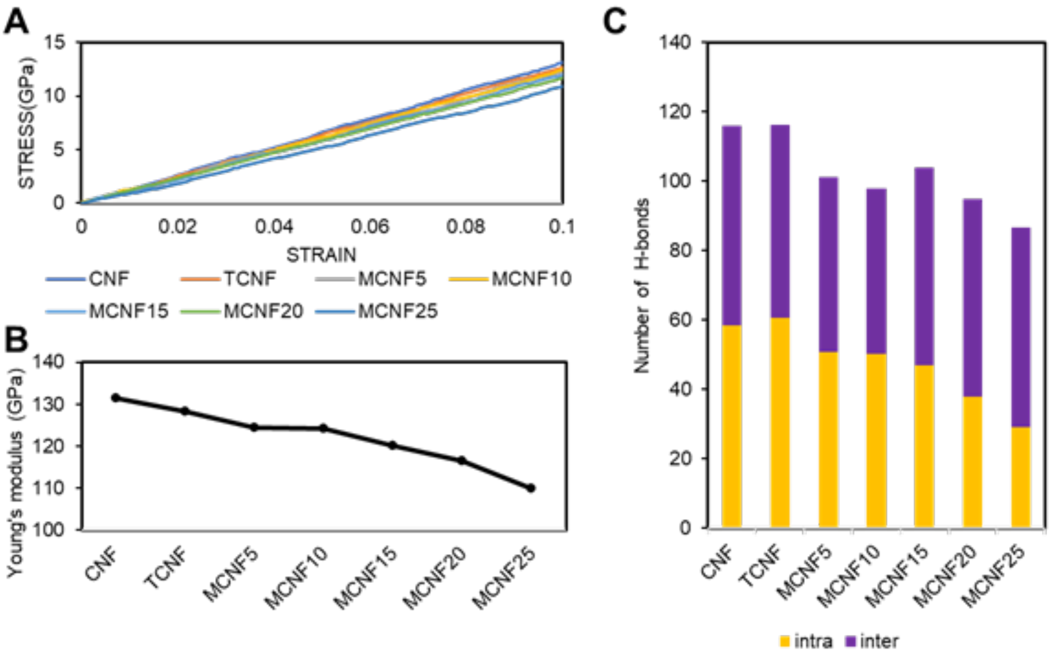

Fig. S4. (A) Young's modulus of tensile strength of each modified nanocellulose. (B) Stress-strain curves of various modified cellulose nanofibers under tensile stress. (C) Hydrogen bond composition of CNFs in vacuum.

V. Detail of the assessment of equilibrium and comparison of the densities for GC/MCNF hydrogel systems.

The system's equilibrium state was determined using root-mean-square deviation (RMSD). As shown in Figure S5(A), the hydrogel system reached stability after 70 ns. Additionally, the variations in end-to-end distance and radius of gyration over time for cellulose nanofibers with different degrees of modification were analyzed, as shown in Figures S5(B) and S5(C). Since the significant fluctuations in CNF values within the hydrogel ceased after 70 ns, we conclude that the models in both environments successfully reached equilibrium.

After equilibrium simulations, the density of the hydrogel model fell within around 1 g/cm3, which is consistent with the reported values in experimental studies,[48, 49], as shown in Figures S6, indicating the reliability of our simulation approach in investigating the spatial structure and interactions within the system.

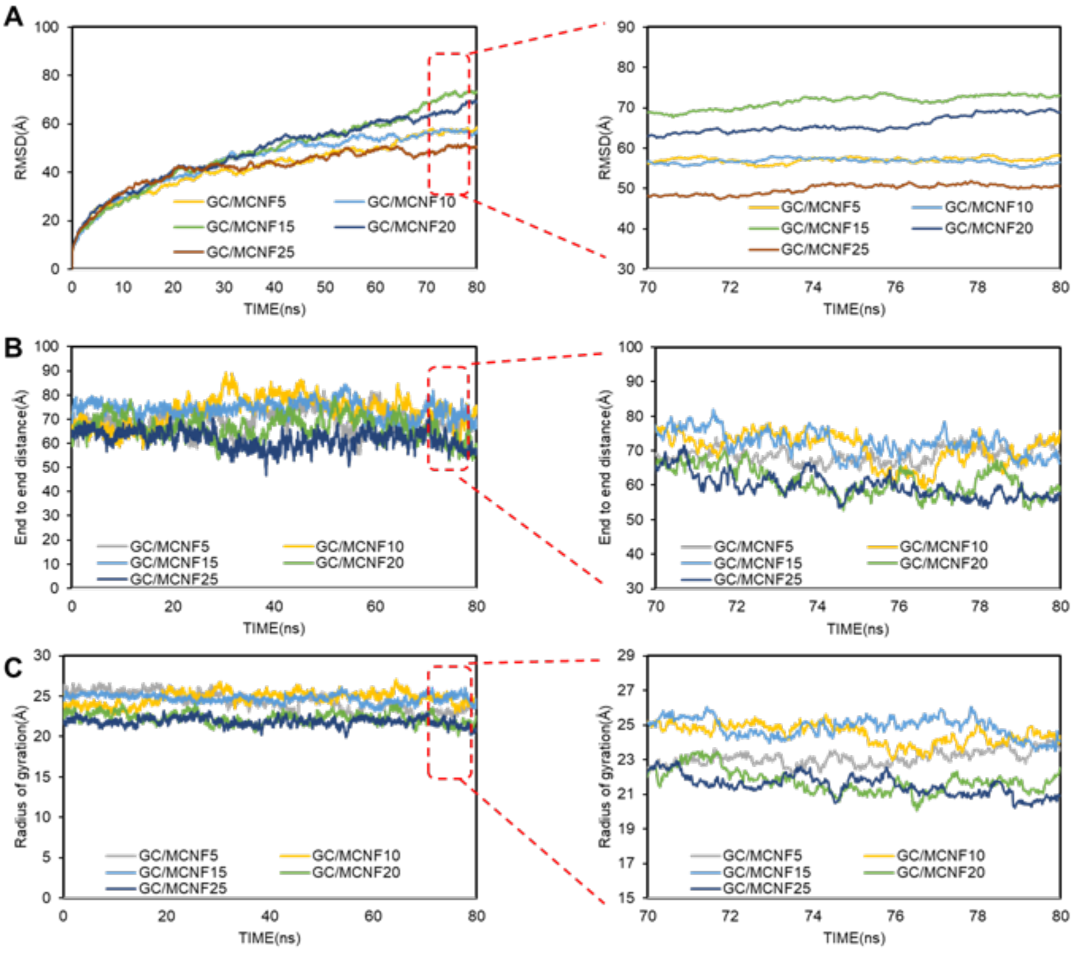

Figure S5. (A) RMSD, (B) end-to-end distance, and (C) radius of gyration of MCNF in hydrogels as a function of the degree of modification. The left figure shows the changes in these values during the simulation, and the right figure shows the changes during the equilibrium stage.

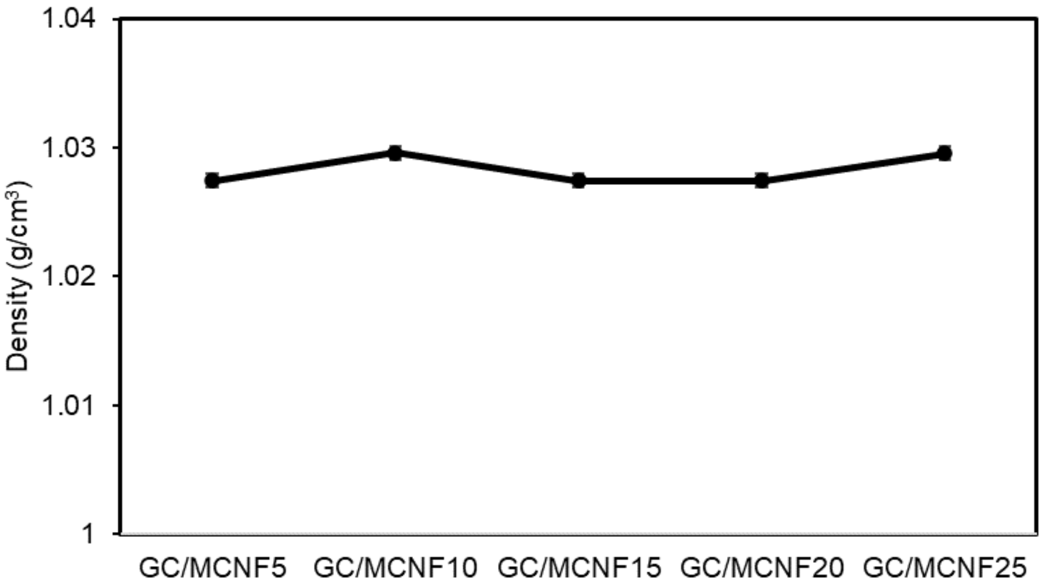

Figure S6. Density of the hydrogel system of MCNF with different aldehyde modification degrees.

VI. Detailed analysis of the end-to-end distance for the GC/MCNF hydrogel systems.

A more detailed analysis of the end-to-end distance of the GC/MCNF hydrogel System is conducted to understand the significance of the results better. Under the same degree of aldehyde substitution, the results of each chain of MCNF will not be much different. Error bars are standard deviations, calculated from 500 data points over 10 ns in the analysis. The end-to-end distance values of glycol chitosan are mostly in the range of 40Å to 60Å, and it can be observed that the end-to-end distance distribution of glycol chitosan shifts to the left when the degree of aldehyde substitution increases.

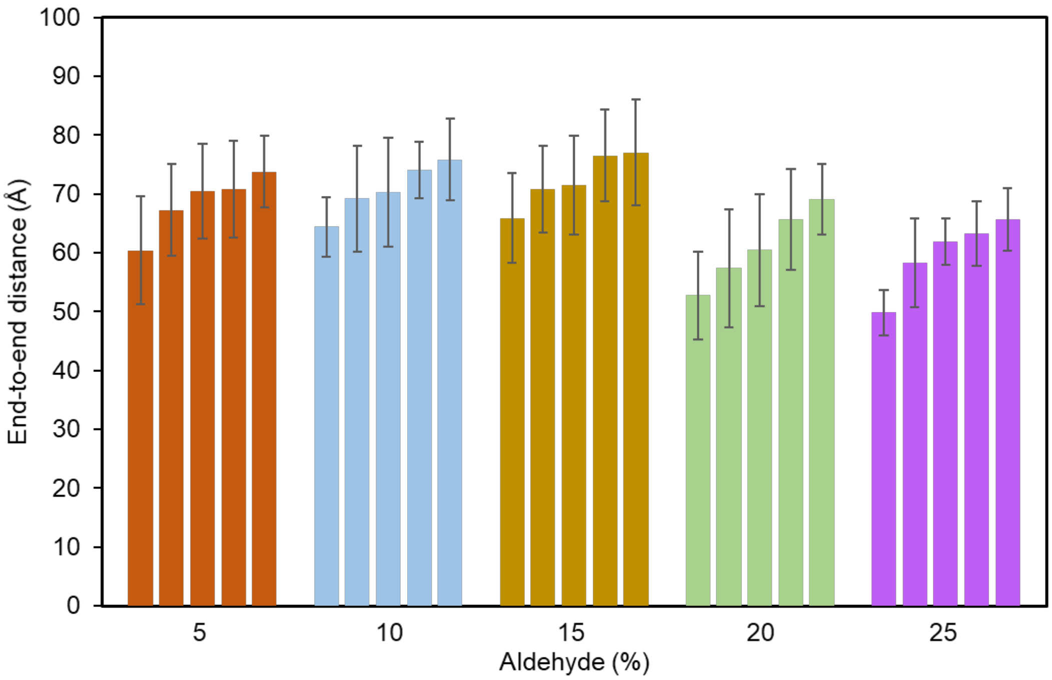

Fig. S7. The end-to-end distance of each MCNF molecular chain for the GC/MCNF hydrogels System.

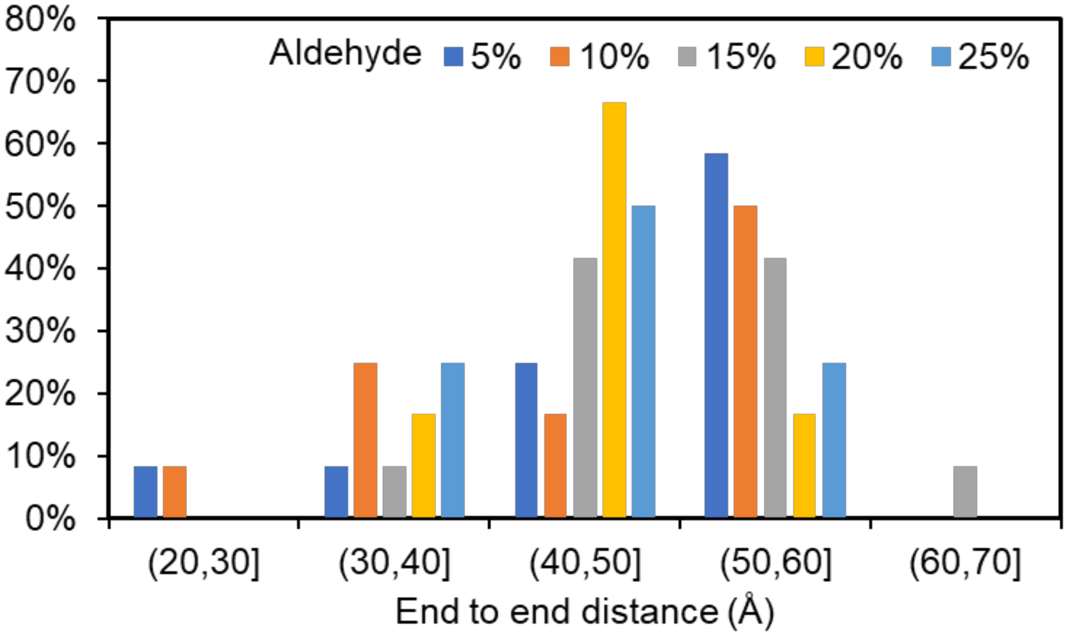

Fig. S8. Quantitative distribution of end-to-end distances of glycol chitosan in GC/MCNF hydrogels System.

VII. Comparison of results before and after Schiff base modeling.

We performed Schiff base modeling and simulations for sites where the distance between amine and aldehyde groups is less than 4 Å, and the results are shown in Figure S9. The Schiff base had little effect on the end-to-end distance and the number of hydrogen bonds in the hydrogel systems.

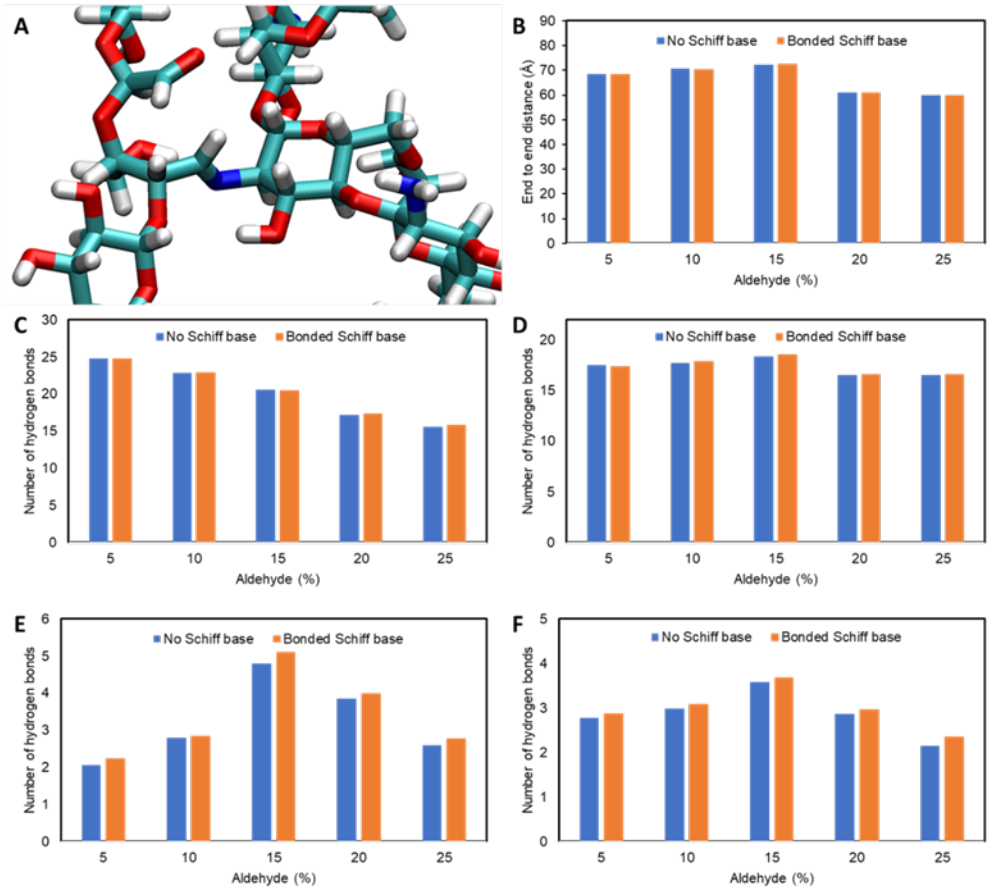

Fig. S9. (A) Schematic diagram of Schiff base bond model. (B) Comparison of end-to-end distance results with and without the Schiff base modeling. Comparison of (C) MCNF intramolecular (D) MCNF intermolecular (E) GC-MCNF (F) GC intermolecular hydrogen bond number results with and without the Schiff base modeling.

References

(19) Gupta, A.; Khodayari, A.; van Duin, A. C. T.; Hirn, U.; Van Vuure, A. W.; Seveno, D. Cellulose Nanocrystals: Tensile Strength and Failure Mechanisms Revealed Using Reactive Molecular Dynamics. Biomacromolecules 2022, 23 (6), 2243-2254. DOI: 10.1021/acs.biomac.1c01110.

(20) Wu, X.; Moon, R. J.; Martini, A. Tensile strength of Iβ crystalline cellulose predicted by molecular dynamics simulation. Cellulose 2014, 21 (4), 2233-2245. DOI: 10.1007/s10570-014-0325-0.

(43) Zhai, L.; Kim, H. C.; Kim, J. W.; Kang, J.; Kim, J. Elastic moduli of cellulose nanofibers isolated from various cellulose resources by using aqueous counter collision. Cellulose 2018, 25 (7), 4261-4268. DOI: 10.1007/s10570-018-1836-x.

(44) Eichhorn, S. J.; Davies, G. R. Modelling the crystalline deformation of native and regenerated cellulose. Cellulose 2006, 13 (3), 291-307. DOI: 10.1007/s10570-006-9046-3.

(45) Ketoja, J.; Paavilainen, S.; McWhirter, J. L.; Róg, T.; Järvinen, J.; Vattulainen, I. Mechanical properties of cellulose nanofibrils determined through atomistic molecular dynamics simulations. 2012, 27 (2), 282-286. DOI: doi:10.3183/npprj-2012-27-02-p282-286 (acccessed 2025-03-17).

(46) Khodayari, A.; Van Vuure, A. W.; Hirn, U.; Seveno, D. Tensile behaviour of dislocated/crystalline cellulose fibrils at the nano scale. Carbohydrate Polymers 2020, 235, 115946. DOI: https://doi.org/10.1016/j.carbpol.2020.115946.

(47) Dri, F. L.; Hector, L. G.; Moon, R. J.; Zavattieri, P. D. Anisotropy of the elastic properties of crystalline cellulose Iβ from first principles density functional theory with Van der Waals interactions. Cellulose 2013, 20 (6), 2703-2718. DOI: 10.1007/s10570-013-0071-8.

(48) RANPATI, D. H. I. Ultrasound-stimulated behaviors on biomass hydrogel medicines for drug release applications. 2022.

(49) Kopač, T.; Abrami, M.; Grassi, M.; Ručigaj, A.; Krajnc, M. Polysaccharide-based hydrogels crosslink density equation: A rheological and LF-NMR study of polymer-polymer interactions. Carbohydrate Polymers 2022, 277, 118895. DOI: https://doi.org/10.1016/j.carbpol.2021.118895.
